# A Unusual Twist: Cecal Lymphangioma as an Unusual Culprit in Adult Intussusception

**DOI:** 10.7759/cureus.92231

**Published:** 2025-09-13

**Authors:** Daniela Avila, Natalie Nagib, Chinwe Okonkwo, Armin Kamyab

**Affiliations:** 1 College of Medicine, Lake Erie College of Osteopathic Medicine, Bradenton, USA; 2 Family Medicine, AdventHealth Sebring, Sebring, USA; 3 General Surgery, AdventHealth Sebring, Sebring, USA

**Keywords:** adult intussuception, cecal lymphangioma, cecal ulcer, intussuception, lymphangioma, right hemicolectomy

## Abstract

Lymphangiomas are uncommon, benign lesions of the lymphatic system that most often appear in childhood. In adults, particularly within the gastrointestinal tract, they are seen far less frequently. Cecal lymphangiomas are especially rare and are not commonly recognized as a lead point in adult intussusception. We present the case of a previously healthy 41-year-old man who developed ileocecal intussusception due to a cecal lymphangioma. This case highlights the importance of considering unusual benign etiologies when evaluating adult intussusception.

## Introduction

Intussusception occurs when one segment of the bowel telescopes into another, leading to obstruction and potential ischemia [1]. While common in children, non-transient adult intussusception is rare, accounting for fewer than one in 1,300 abdominal surgeries, and is usually secondary to an underlying obstructive pathology, malignancy being the most frequent etiology [1,2]. Benign causes, however, are occasionally identified, and lymphangiomas represent one of the rarest. [1,2].

Lymphangiomas are benign malformations of the lymphatic system, typically congenital but occasionally acquired in adults due to trauma, inflammation, or obstruction [3]. They are rare in the gastrointestinal tract, particularly in the cecum, and even less frequently serve as a lead point for intussusception [4,5]. With increased colonoscopy use, incidental detection has risen, though symptomatic cases remain uncommon [6]. With this background in mind, we present the case of an adult patient who developed intussusception secondary to a colonic lymphangioma.

## Case presentation

A 41-year-old otherwise healthy man presented to the emergency department with a one-day history of acute onset, crampy right-sided abdominal pain. The patient stated he had recently begun using a new pre-workout supplement (C4 Sport Performance Blend) and a post-workout recovery supplement (Urolithin-A). Only medication was the occasional use of non-steroidal anti-inflammatory drugs (NSAID) for workout-related soreness, reportedly taken one to two times per week. 

Abdominal examination revealed mild generalized tenderness, most pronounced in the right lower quadrant, with mild guarding but without rebound tenderness or rigidity.

**Figure 1:**
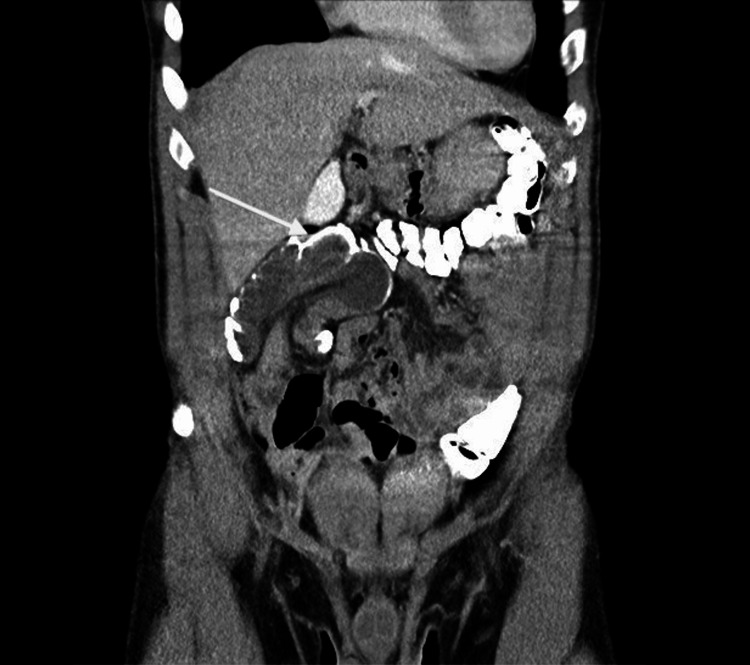
Coronal CT abdomen with IV contrast shows persistent large ileocolic intussusception.

**Figure 2:**
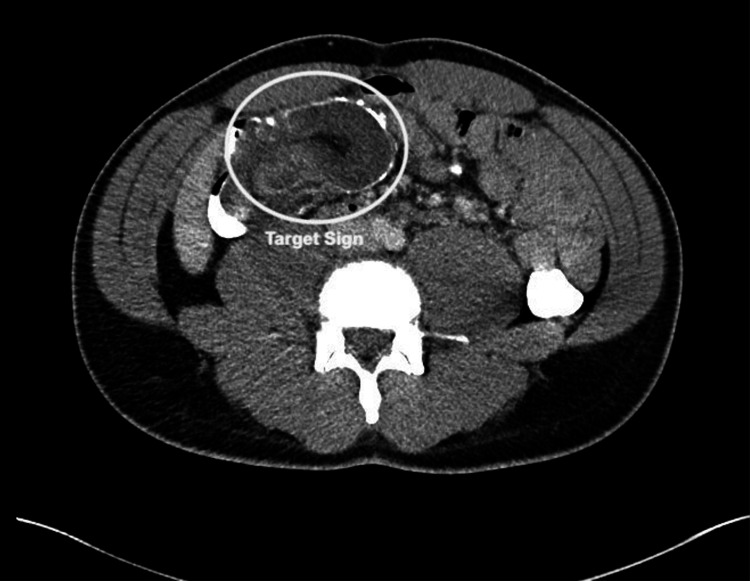
Axial CT scan of the abdomen with IV contrast showing the classic "target sign" of ileocolic intussusception.

A computerized tomography (CT) scan revealed an ileocolic intussusception. An interval CT scan was performed to rule out common, transient intussusception, which is often observed in adults. A follow-up CT, 24 hours after the first scan, confirmed persistent ileocolic intussusception, suggesting a structural lead point (Figures 1, 2).

**Figure 3:**
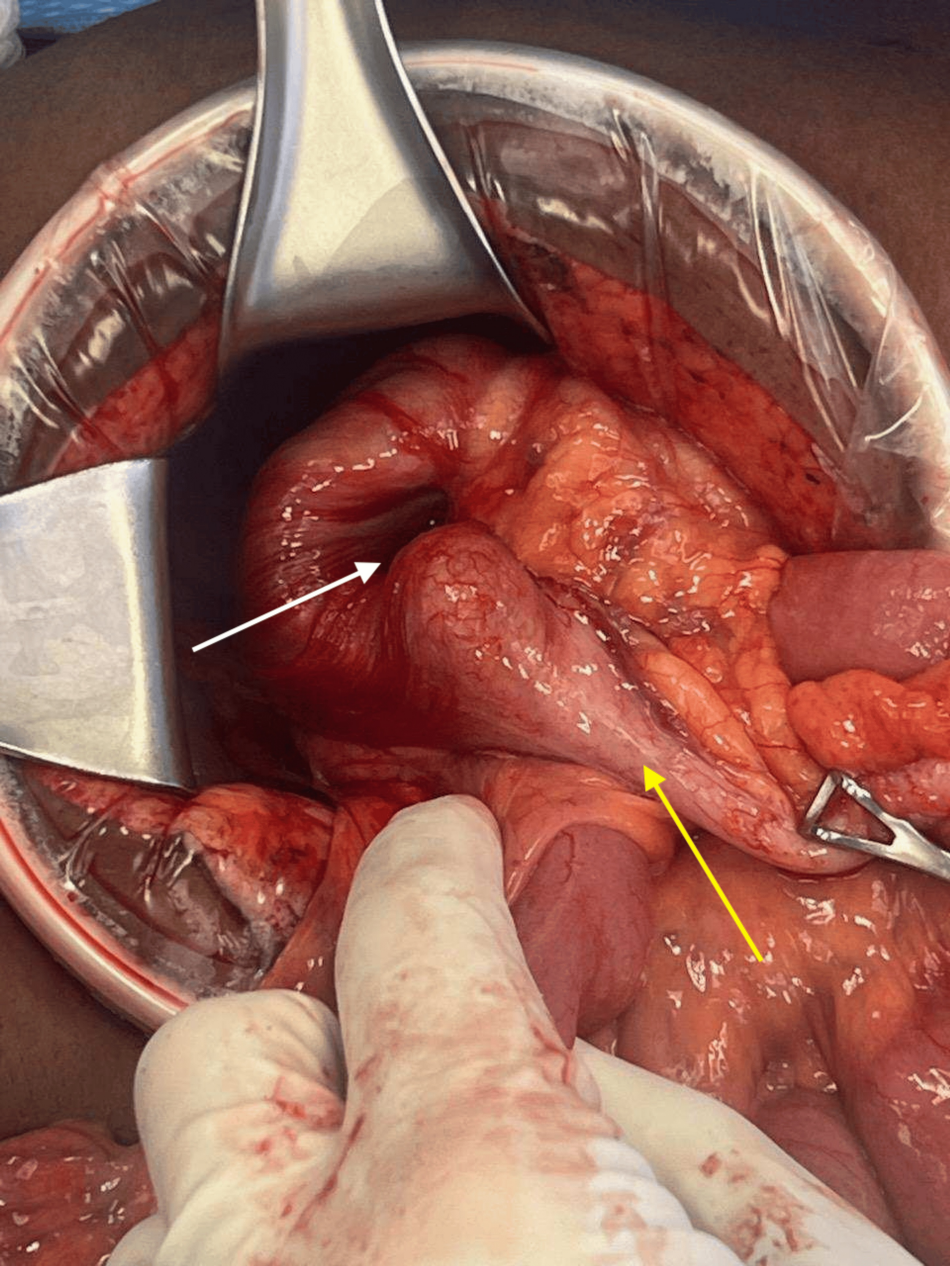
Intraoperative photo showing intussuscepted ileum (yellow arrow) into mid-ascending colon (white arrow).

The decision was therefore made to proceed with surgical intervention, and the patient underwent a right hemicolectomy. Intraoperative findings confirmed intussusception of the ileum into the ascending colon, with an irregularly shaped cecum that was suspected to be the lead point (Figure 3).

The patient tolerated the procedure well. His recovery was uneventful. On postoperative day 3, he was discharged home.

Histopathological analysis revealed ulcerated colonic mucosa with marked acute inflammation and necrosis. A benign submucosal proliferation of lymphatic spaces, consistent with lymphangioma, was identified. Additional findings included focal ischemic-type mucosal injury, serosal adhesions, reactive lymph nodes, and an appendix with fibrous obliteration of the tip. No evidence of dysplasia or malignancy was detected, and all resection margins were viable.

## Discussion

Refractory or persistent intussusception in adults is an uncommon condition, typically associated with a pathological lead point. While most adult intussusception cases are linked to malignancy, rare instances of benign etiologies, such as cecal lymphangioma, have been reported [5-8]. Similar cases of cecal lymphangioma causing adult intussusception have been reported, most involving women in their 30s-40s who presented with abdominal pain and required surgical resection [6-8]. In contrast, other cecal lymphangiomas have been detected incidentally on colonoscopy and managed conservatively without surgery [5]. This case adds to the limited literature by presenting a middle-aged male patient with a cecal lymphangioma as the lead point for intussusception, an unusual presentation of this benign entity.

The most common type of adult intussusception is colocolic (16.82%), followed by enteric (13.28%), ileocolic (4.89%), and ileocecal (0.78%) and is typically secondary to a structural abnormality rather than idiopathic, as seen in pediatric cases [2,9,10]. Dysmotility and mechanical factors contribute to the invagination process.

Notably, ileocolic intussusception in adults is often associated with malignancy, making this case of benign lymphangioma an atypical clinical presentation. A pathological lead point is identified in approximately 44.8% of cases, with colocolic intussusception carrying the highest malignancy risk [2,10-12]. Surgical intervention remains the primary treatment, particularly for colonic intussusception, due to high rates of malignancy is a major concern [2,13].

Lymphangiomas are congenital malformations of the lymphatic system resulting from aberrant lymphatic sequestration. While typically identified in childhood, they can develop in response to trauma, chronic inflammation, or lymphatic obstruction [14]. The mechanism by which a lymphangioma leads to intussusception remains speculative, but localized lymphatic disruption may contribute to tissue edema and abnormal peristalsis, facilitating bowel telescoping [15]. Although complete resection is typically curative, recurrence rates vary from 0% with full excision to nearly 100% with incomplete removal, reinforcing the need for post-operative colonoscopy and surveillance [15,16].

The presence of a cecal lymphangioma as a lead point for intussusception makes this case particularly novel, given its location. The lymphangioma in this patient was in the cecum, an uncommon site for such lesions. Intraabdominal lymphangiomas represent less than 5% of all lymphangiomas and have been reported in various locations, including the mesentery, retroperitoneum, omentum, and colon [6]. With increased colonoscopy use, incidental detection and diagnosis of colonic lymphangiomas have risen [6].

A solitary cecal ulcer was also identified in this patient. Although in this case the ulcer may have been due to the intussusception, it does raise questions about its possible role in the pathogenesis of intussusception. Solitary cecal ulcers are also rare and can result from infections, inflammatory bowel disease, NSAID use, or ischemia [11,17]. In this case, the ulcer could have weakened the bowel wall, predisposing it to intussusception, or it may have developed secondarily due to ischemia from prolonged obstruction. Histopathological findings of necrosis support the latter hypothesis, suggesting the ulcer was potentially a consequence of the intussusception [1]. Notably, no reported cases describe cecal ulcers coexisting with both intussusception and lymphangiomas, highlighting a rare confluence of benign pathologies and the need for further study into their interplay.

## Conclusions

Adult intussusception remains a rare and diagnostically challenging condition, often linked to underlying structural or pathological causes, most commonly malignancy. This case underscores the importance of maintaining a broad differential diagnosis, particularly when imaging suggests persistent intussusception. While cecal lymphangiomas are rare, they can act as a benign lead point, as demonstrated in this patient. The coexistence of a solitary cecal ulcer, although likely ischemic in origin, presents a unique aspect that has not been previously documented in combination with lymphangioma-induced intussusception. Surgical resection remains the cornerstone of management, and given the possibility of recurrence or missed pathology, postoperative colonoscopic surveillance should be strongly considered. Ultimately, this case contributes to the limited body of literature on benign causes of adult intussusception and highlights the need for heightened clinical suspicion, especially in atypical presentations.
